# MITIGATING LONG-TERM CONSEQUENCES OF COVID-19 ON OLDER ADULTS IN HONG KONG, SINGAPORE, THAILAND, AND THE US

**DOI:** 10.1093/geroni/igac059.1079

**Published:** 2022-12-20

**Authors:** Ladda Thiamwong, Vivian Wei Qun Lou

## Abstract

The impact of COVID-19 on older adults goes beyond a higher risk for serious infection to long-term consequences such as decreased well-being and disability. In 2020, we formed an international and interdisciplinary research group and adopted a biopsychosocial model as a conceptual framework to guide our collaboration. Our ultimate goal is to mitigate the long-term health consequences of the pandemic and utilize affordable technology-based interventions to enhance the quality of life for older adults in Hong Kong, Singapore, Thailand, and the US. Five empirical studies will be presented with topics ranging from quality of life, fear of falling, attitudes toward technology, and the utilization of technology-based interventions. A path analysis from the first study indicated that physical frailty was the strongest direct power on quality of life followed by depressive symptoms, and life-space mobility. The second study found that fear of falling was predicted by falls risk, resistance, COVID fear, and health conditions. The third study revealed a strong connection between the COVID severity and positive attitudes toward technology, and social support facilitates the adoption of technology. The fourth study reported the benefits of using a technology-based body-mind intervention to alter a mismatch between fear of falling and actual fall risk and to increase the accessibility of the fall intervention. The final study indicated that utilizing an alternative online exercise program during the pandemic increased exercise regularity and enhanced motivation. Together, all five studies contribute to strategic implications on global mitigating long-term health consequences and essential issues for further research.

